# Recent Advances in Cyanamide Chemistry: Synthesis and Applications

**DOI:** 10.3390/molecules22040615

**Published:** 2017-04-12

**Authors:** M. R. Ranga Prabhath, Luke Williams, Shreesha V. Bhat, Pallavi Sharma

**Affiliations:** School of Chemistry, Joseph Banks Laboratories, University of Lincoln, Lincoln LN6 7DL, UK; ranga_prabhath@yahoo.com (M.R.R.P.); luwilliams@lincoln.ac.uk (L.W.); shreeshabhat@yahoo.co.in (S.V.B.)

**Keywords:** cyanamide, synthesis, aminocyanation, cycloaddition, electrophilic cyanation, radical reaction, coordination chemistry

## Abstract

The application of alkyl and aryl substituted cyanamides in synthetic chemistry has diversified multi-fold in recent years. In this review, we discuss recent advances (since 2012) in the chemistry of cyanamides and detail their application in cycloaddition chemistry, aminocyanation reactions, as well as electrophilic cyanide-transfer agents and their unique radical and coordination chemistry.

## 1. Introduction

Cyanamide enjoys a rich chemical history, which can be traced to its unique and chemically promiscuous nitrogen-carbon-nitrogen (NCN) connectivity. The chemistry of the nitrile-substituted amino-group of the ‘cyanamide-moiety’ is dominated by an unusual duality of a nucleophilic *sp*^3^-amino nitrogen and an electrophilic nitrile unit.

The reported use of unsubstituted cyanamide (NH_2_CN) and metal cyanamides (MNCN, where M = metal) date back as far as the late 19th century, where the likes of calcium cyanamide (CaNCN) was used as a fertilizer, and later as source of ammonia and nitric acid, which fueled the industrial production of metal cyanamides. In contrast, the reported use of the corresponding substituted organic cyanamides (RNHCN or RR’NCN) gathered pace only in more recent years. 

The last decade has witnessed a significant increase in the use and application of substituted cyanamides in synthetic chemistry, along with development of more sustainable and robust synthetic routes to these important family of compounds.

A number of excellent review articles describing the chemistry of cyanamide have appeared in the literature in recent years, and we encourage the readers to investigate these articles. Nekasarov and co-workers [1] summarized the synthesis and reactions of mono and disubstituted cyanamides in a seminal review article in 2004, whereas the literature from 2004 to 2012 was captured concisely by Fensterbank and Lacôte et al [2]. Thus, in the present review, we limit our discussion to developments appearing in the literature from 2012. The present review is by no means exhaustive and we apologize to authors whose work has been omitted. The present article focuses on some leading topics in cyanamide chemistry such as aminocyanation, electrophilic cyanation, selected cycloaddition reactions, coordination chemistry and radical reactions, whereas more traditional chemistry has not been covered here and we would like to direct the readers to appropriate primary literature.

## 2. Synthesis of Mono and Disubstituted Cyanamides

The direct alkylation of metal cyanamides such as calcium cyanamide is the most straightforward approach to mono and disubstituted derivatives, and reports on these strategies have been previously covered [1,2,3]. This section details more recent approaches, including development of new reagents to access substituted cyanamides.

### 2.1. N-Cyanation of Secondary Amine

#### 2.1.1. Using Electrophilic Nitrile [CN]^+^ Reagents

The electrophilic cyanation of secondary amines is the most prevalent method used to access disubstituted cyanamides, with cyanogen bromide (BrCN) being the most common reagent. Though cheap and effective, the toxicity associated with this reagent has prompted the development of safer cyanating agents such as 2-cyanopyridazin-3(2*H*)-one, cyanobenzimidazole, and 1-cyanoimidazole [4,5,6].

**Scheme 1:**
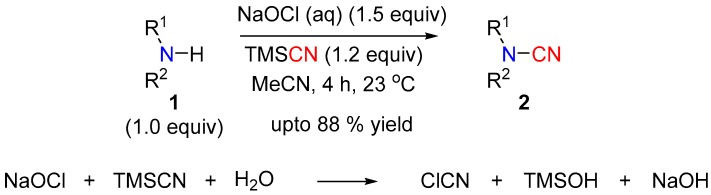
In situ generation of CNCl for cyanation.

In 2014, Chen et al. used a combination of hypochlorite and TMSCN for the in situ generation of cyanogen chloride (CNCl) to accomplish the *N*-cyanation of a variety of secondary amines [7]. The hypochlorite oxidizes the TMSCN to release the active CNCl reagent, which then cyanates nucleophilic amines. Both dialkyl and alkyl-aryl amines were readily cyanated using this method, offering the products in good to excellent yields (Scheme 1).

**Scheme 2:**
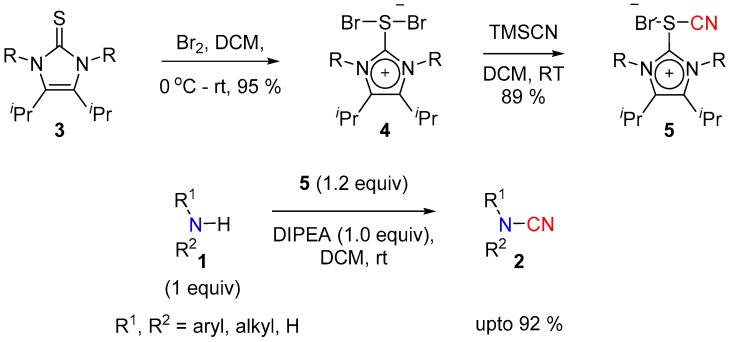
Thiocyanoimidazolium salts for electrophilic cyanation.

Alcarazo and co-workers have developed the thiocyanoimidazolium salt **5** as an electrophilic cyanation reagent for secondary amines [8]. Halogenation of thiourea led to the hypervalent sulfur species **4**, which upon treatment with TMSCN provided the active reagent **5**. In the presence of an amine base such as diisopropylethylamine (DIPEA), the reagent **5** successfully transferred the active [CN]^+^ to a number of alkyl and aryl *sec*-amines to give corresponding cyanamides in good yield (Scheme 2). The protocol was deemed to be operationally simple and wide in scope, however precaution must be taken when handling the cyanating reagent **5**; just like the isolobal hypervalent iodine reagents, they show strong exothermic decomposition upon heating.

**Scheme 3:**
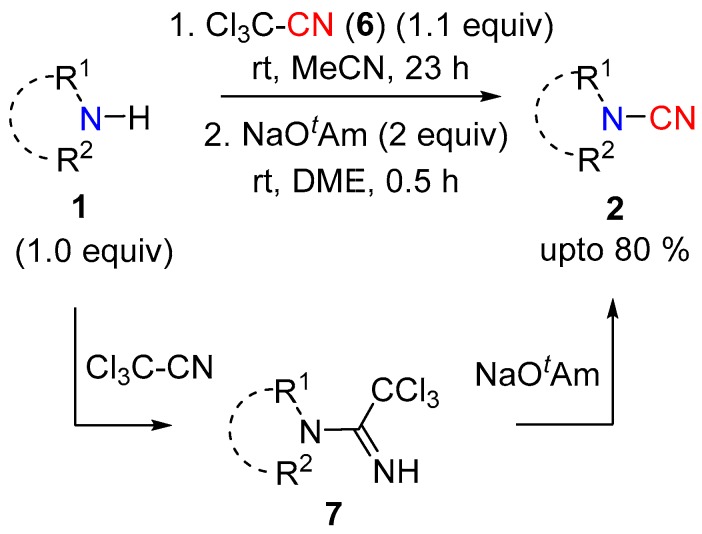
Trichloroacetonitrile for cyanation.

The Morrill group reported trichloroacetonitrile (**6**) as an effective cyanating reagent of *sec*-amines following a one-pot protocol [9]. Though not strictly a [CN]^+^ transfer reagent, the two-step-single-pot protocol involved formation of the amidine **7** (Scheme 3) via nucleophilic addition and its further conversion into the nitrile mediated by NaO*^t^*Am base (Scheme 3). Both cyclic and acyclic dialkyl cyanamides were obtained in good yields. The trichloroacetonitrile reagent **6** allowed the selective cyanation of secondary amines in the presence of other nucleophilic centers, which is a significant benefit compared to other cyanating reagents.

#### 2.1.2. Under Copper Catalysis

**Scheme 4:**
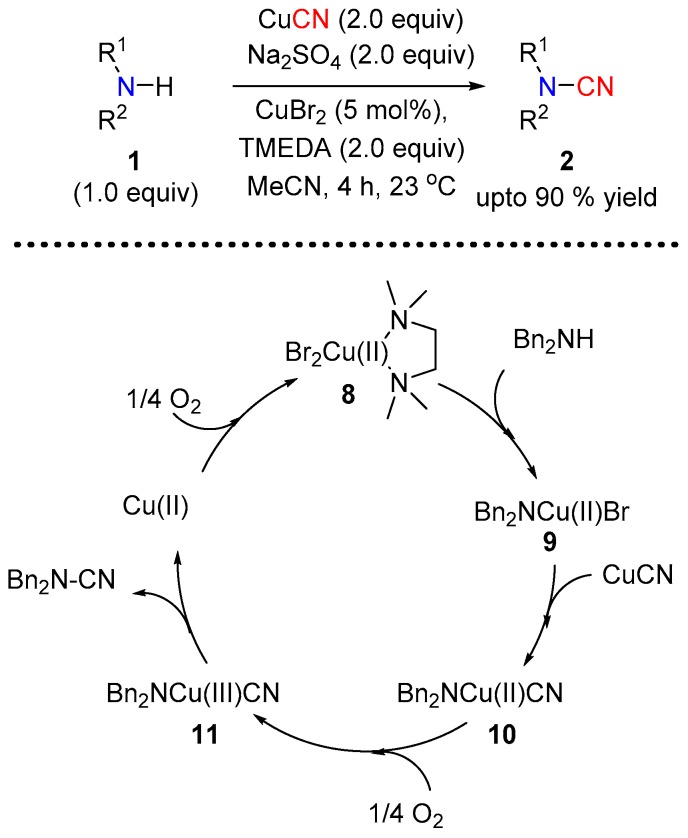
Copper catalyzed cyanation of secondary amines.

Cheng et al. developed a transition metal catalyzed cyanation of *sec*-amines for N–CN bond formation under oxidative coupling conditions [10], using CuCN as the source of nitrile. When used in combination with *N*,*N*,*N*′,*N*′-tetramethylethylenediamine (TMEDA), which itself was proposed to play the dual role of a ligand and a base in the catalytic cycle, molecular oxygen (O_2_) and sodium sulfate, the *sec*-amine underwent cyanation in the presence of CuBr_2_ (Scheme 4). The CuCN was also shown to self-catalyze the cyanation reaction when conducted in the absence of any additional copper source. Based on the experimental evidence the authors proposed the catalytic cycle illustrated in Scheme 4, where a Cu(II) species is thought to participate in the catalytic cycle.

**Scheme 5:**
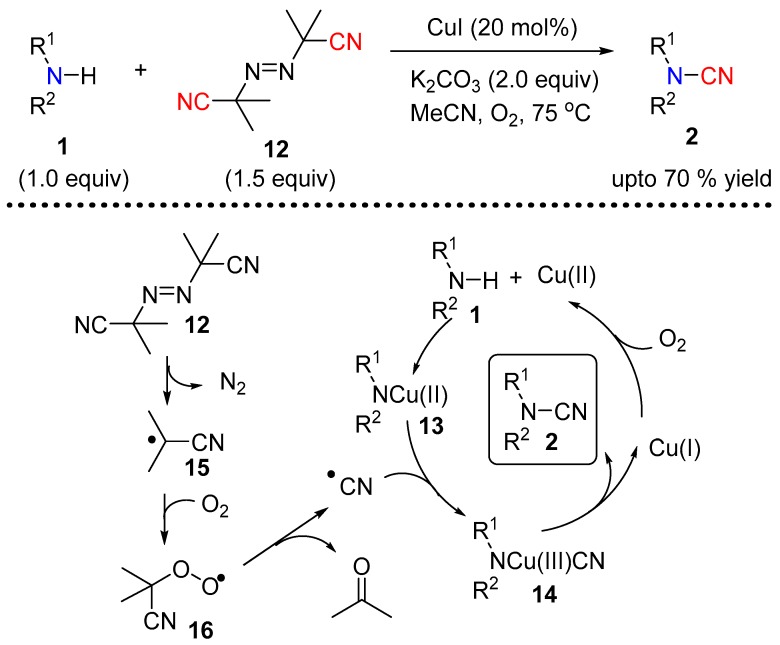
AIBN mediated cyanation of secondary amine.

Continuing their efforts to provide a safer source of cyanide for N–CN bond formation under copper catalysis, the authors investigated the use of the radical initiator azobisisobutyronitrile (**12**, AIBN) [11]. In the presence of CuI, in combination with molecular oxygen and K_2_CO_3_, AIBN efficiently led to a variety of disubstituted cyanamides starting from both alkyl and aryl secondary amines (Scheme 5). Based on their experimental observations the authors proposed a radical pathway, which incorporates the generation of a nitrile radical from AIBN. Molecular oxygen mediated oxidation of Cu(I) to Cu(II) leads to species **13** in the presence of the base. The generation of nitrile radical from AIBN assisted by molecular oxygen insert into **13** to give **14**. A reductive elimination of **14** provides the final *N*-cyanated product **2** (Scheme 5).

### 2.2. Cyanamides from Amidoximes and Guanidoximes

**Scheme 6:**
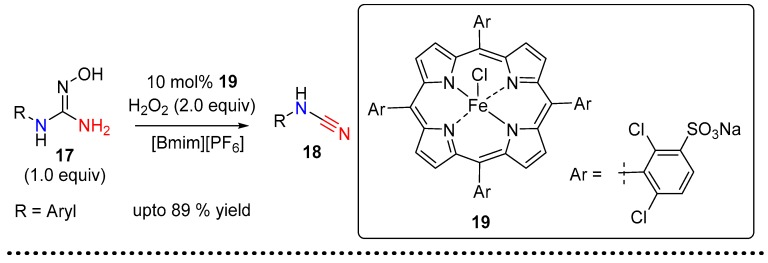

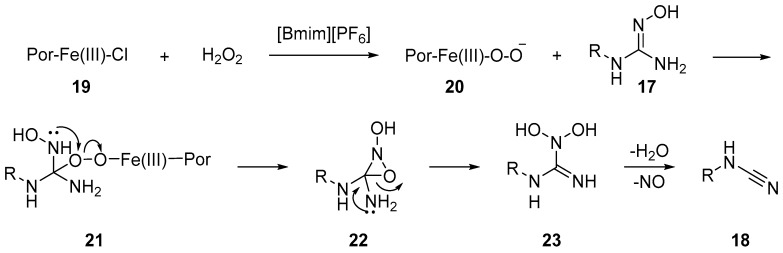
Conversion of guanidoximes to monosubstituted cyanamide.

Chauhan and co-workers reported the synthesis of monosubstituted *N*-arylcyanamides via the oxidation of the corresponding guanidoximes in an ionic liquid (Scheme 6) [12], which is proposed to mimic the natural enzymatic oxidation of oximes to nitriles. The imidazolium ionic liquid was used as the reaction medium for the H_2_O_2_ mediated oxidation catalyzed by the water soluble anionic iron(III)-porphyrins [TAPS_4_Fe(III)Cl].

**Scheme 7:**
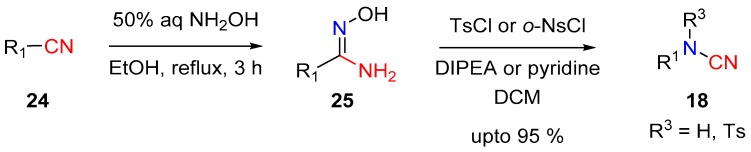
Amidoximes to substituted cyanamides.

A peroxo-species like **21** was proposed as an intermediate, itself obtained upon nucleophilic addition of iron-peroxo **20** on the oxime double bond. The peroxo-complex **21** then undergoes cyclization followed by selective ring opening to give **23**, followed by the elimination of water and loss of NO to render the target cyanamide **18** (Scheme 6).The generation of cyanamide type species from the reaction of amidoxime with aryl-sulfonyl-chloride was first observed by Tiemann in 1891 [13]. In their seminal report, Tiemann detected a series of diverse mixture of products, which included cyanamide, carbodiimide, guanidine and benzimidazole, among others. In 2014 Chien et al. revisited this reaction and developed conditions that led to the selective formation of monosubstituted aryl-cyanamide from the corresponding aryl-amidoxime [14]. Upon treatment with *p*-TsCl in the presence of DIPEA or pyridine, the amidoximes were converted to cyanamides in good to excellent yields (Scheme 7). The authors observed that the reaction of the amidoxime **25** with *p*-TsCl, and the subsequent product distribution, was dependent on the electronics of the substrates. It was observed that cyanamide products were predominantly formed with electron rich aryl substrates. The authors further observed that the rate of the key rearrangement step depends on the crucial N–O bond cleavage and reports *o*-NsCl as a better leaving group for challenging substrates (electron-deficient aryl- and alkyl-substrates) at elevated reaction temperatures. A variety of aryl, alkyl and vinyl cyanamides can be accessed under these conditions.

### 2.3. Cyanamides from Isoselenocyanates

**Scheme 8:**
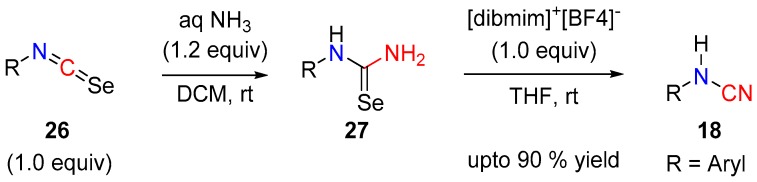
Isoselenocyanates to aryl-cyanamide.

The dehydration of urea or desulfurization of thiourea are both well-established methods that are used to access cyanamides [2]. Similarly, cyanamides can also be prepared by the deselenization of selenourea. In 2015, Xie and co-workers, described a one-pot hypervalent iodine-mediated deselenization of aryl-isoselenocyanates [15], using the recyclable ionic liquid supported 1-(4-diacetoxyiodobenzyl)-3-methylimidazolium tetrafluoroborate ([dibmim]^+^[BF_4_]^–^), which gave a variety of cyanamides (Scheme 8).

**Scheme 9:**
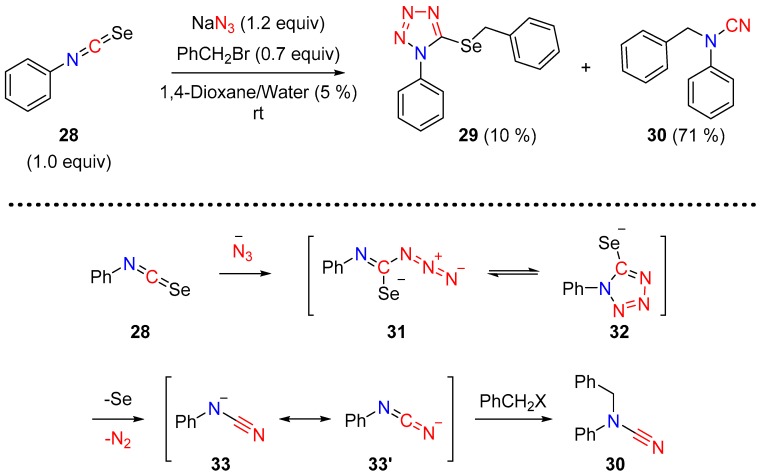
Synthesis of disubstituted cyanamide by the cycloaddition of azide with isoselenocyanate.

During the syntheses of 5-alkylselanyl-1-aryl-1*H*-tetrazoles from aryl-isoselenocyanates, the serendipitous synthesis of *N*-alkyl-*N*-aryl-cyanamides was observed by Roh and co-workers [16]. The disubstituted cyanamide **30** was formed as predominant product when aryl-isoslelenocyanate **28** was treated with azide followed by an alkyl-halide (Scheme 9). Mechanistically the loss of elemental selenium and dinitrogen (N_2_) from the intermediate tetrazole **32** led to the anionic species **33**, which upon alkylation provided the disubstituted cyanamides (Scheme 9).

## 3. Synthetic Applications of Substituted Cyanamides

### 3.1. Cycloaddition Reactions

The carbon-nitrogen triple bond of cyanamides readily undergo [3 + 2] and [2 + 2 + 2] cycloadditions when exposed to suitable reacting partners, which ultimately lead to the formation of five and six membered ring heterocycles, respectively.

#### 3.1.1. [3 + 2] Cycloaddition

Cyanamides participate as dipolarophiles in [3 + 2] cycloaddition reactions with dipoles to offer cycloadducts; e.g., the reaction with azide dipoles offer 4-aminotetrazole adducts [2]. Recently, cyanamides have been shown to participate in reactions with alkynes and other dipoles to offer a variety of heterocyclic products.

**Scheme 10:**
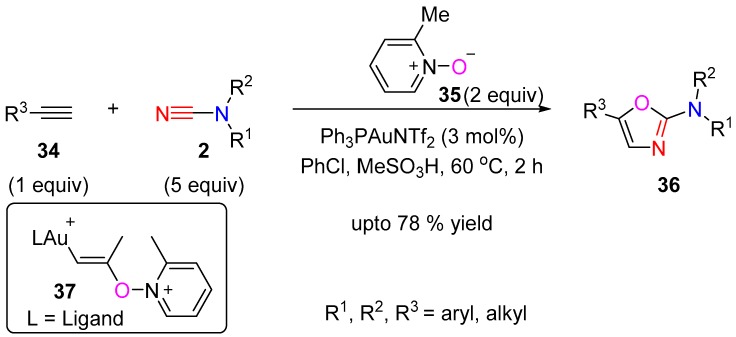
2-Amino-1,3-oxazoles synthesis via cycloaddition of alkynes and cyanamides.

Kukushkhin et al. applied a gold catalyzed heterocyclization of alkynes with substituted cyanamides in the presence of the oxygen donor 2-picoline oxide (**35**), to give 2-amino-1,3-oxazoles **36** (Scheme 10) [17]. A variety of terminal alkynes **34** in the presence of Ph_3_PAuNTf_2_ and 2-picoline oxide in chlorobenzene, underwent cyclization with dimethylcyanamide to offer the 5-substitued 2-amino-1,3-oxazoles **36**. The reaction was proposed to proceed through a β-gold(I)-vinyloxypyridinium intermediate **37**, which is trapped by the cyanamide nitrile.

**Scheme 11:**
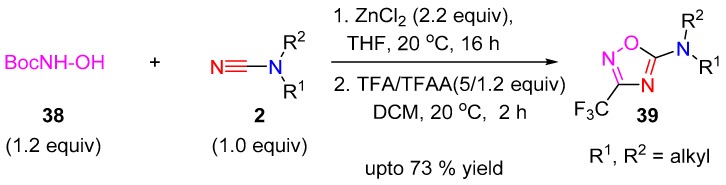
1,2,4-Oxadiazoles synthesis from cyanamides.

A cycloaddition protocol for the synthesis of 1,2,4-oxadiazoles **39** was developed utilizing cyanamides as core building block by Goldberg and co-workers [18]. A zinc-chloride catalyzed reverse nucleophilic addition of *N*-Boc-hydroxylamine (**38**) to the cyanamide-nitrile, followed by acylation, deprotection and ring-closure, mediated by TFAA and TFA, lead to the final cyclized 1,2,4-oxadiazole targets **39** (Scheme 11).

**Scheme 12:**
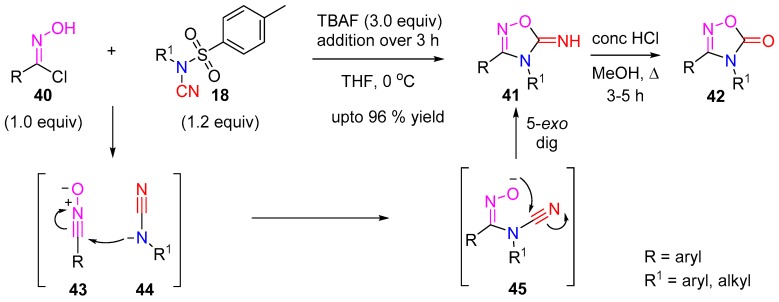
1,2,4-oxadizole-5-imines synthesis from cyanamides.

Recently, we (Sharma and co-workers) investigated the in situ trapping of a cyanamide anion **44** with 1,3-dipoles like nitrile oxide **43** for the syntheses of nitrogen rich heterocycles via a formal [3 + 2] cycloaddition [19]. Using TBAF as a reagent for both the detosylation of *N*-tosyl-cyanamide and the dehydrochlorination of chlorooxime, allowed the formation of both complimentary reactive species in one pot. The nucleophilic addition of the cyanamide anion to the nitrile-oxide followed by 5-*exo*-dig cyclization gave the oxadiazol-5-imine product **41** in excellent yields (Scheme 12). These novel heterocycles, which are useful but somewhat under-represented in pharmaceutical drugs, were further converted to the biologically relevant oxadiazol-5-ones **42** upon acidic hydrolysis. Further investigation into the reactions of cyanamide anion led to the syntheses of other novel heterocycles under trapping conditions with dipoles like nitrile imine.

#### 3.1.2. [2 + 2 + 2] Cycloaddition

The metal-catalyzed [2 + 2 + 2] cyclotrimerization of substituted cyanamides to triazine products are well studied reactions that have been previously reviewed [2]. In contrast, the co-cyclization of the nitrile moiety of cyanamides with dialkyne or alkenyl-nitrile system offers a route to functionally diverse six membered heterocyclic systems such as 2-aminopyridine and aminopyrimidine derivatives.

**Scheme 13:**
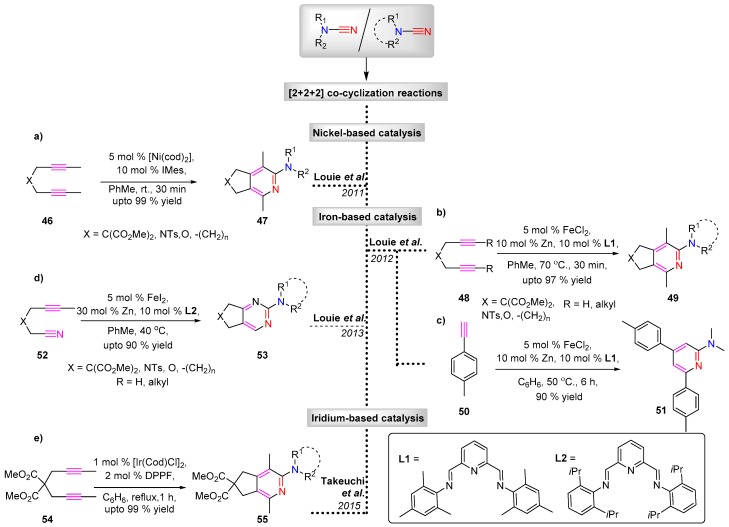
Metal catalyzed [2 + 2 + 2] cycloaddition reactions of cyanamides.

Early reports routinely employed cobalt-based catalysts for such transformations [20,21]. In more recent years nickel, iron and iridium-based catalysts have been applied for the co-cyclization of cyanamides and alkynes to render 2-aminopyridine with excellent regiocontrol. The Louie group reported the use of Ni(cod)_2_ in combination with IMes ligand as an effective catalyst system for the reaction of diyne with a variety of dialkyl cyanamides to give *N*,*N*-disubstituted 2-aminopyridines (Scheme 13a) [22].

Later in 2012, the same group demonstrated the effectiveness of an iron based catalyst system [FeCl_2_/Zn/**L1**] for the cyclization reaction [23]. In such systems, the Fe(II) salt is reduced by Zn to a low valent Fe catalyst species, followed by the slow addition of diyne (Scheme 13b). The authors also report the successful intermolecular cyclization of terminal aryl acetylenes with cyanamides to offer 2-amino-4,6-aryl pyridines **51** (Scheme 13c) [24]. Further modification of the iron based catalyst system was introduced by Wan et al., wherein FeI_2_/dppp/Zn realizes the products in comparable yields and regioselectivity but with the benefit of lower reaction temperatures and catalyst loadings [25].

The [2 + 2 + 2] co-cyclization utilizing cyanamide was further applied by Louie et al. to access aminopyrimidines **53** [26]. The cyclization of alkenyl-nitrile **52** with cyanamide under FeI_2_, *iPr*PDAI, Zn dust in toluene gave access to the substituted pyrimidines (Scheme 13d).

Given the significance and prevalence of substituted aminopyridines in pharmaceutical drugs and natural product cores, more flexible catalyst systems were developed to improve the yield and selectivity. Takeuchi and co-workers found [Ir(cod)Cl]_2_/BINAP as an efficient catalyst for the [2 + 2 + 2] cycloaddition of α,ω-diynes **54** with cyanamide nitriles (Scheme 13e). The authors argue that the presence of a phosphine-ligand-offered flexibility with respect to the catalytic activity, which could be achieved by choosing the appropriate phosphine ligand [27].

### 3.2. N-CN Bond Cleavage

The cleavage of the N–CN bond of cyanamide offers: (i) an electrophilic cyanating agent; (ii) amino-transfer group (aminating reagent); as well as (iii) the synchronized transfer of both amino and nitrile-groups, could offer routes to difunctionalization of C–C multiple bond aminocyanation. These reactions of cyanamides are accomplished under both metal catalysis and metal-free conditions, offering highly functionalized substrates. The present section will detail recent pioneering work in these subcategories.

#### 3.2.1. Aminocyanation

**Scheme 14:**
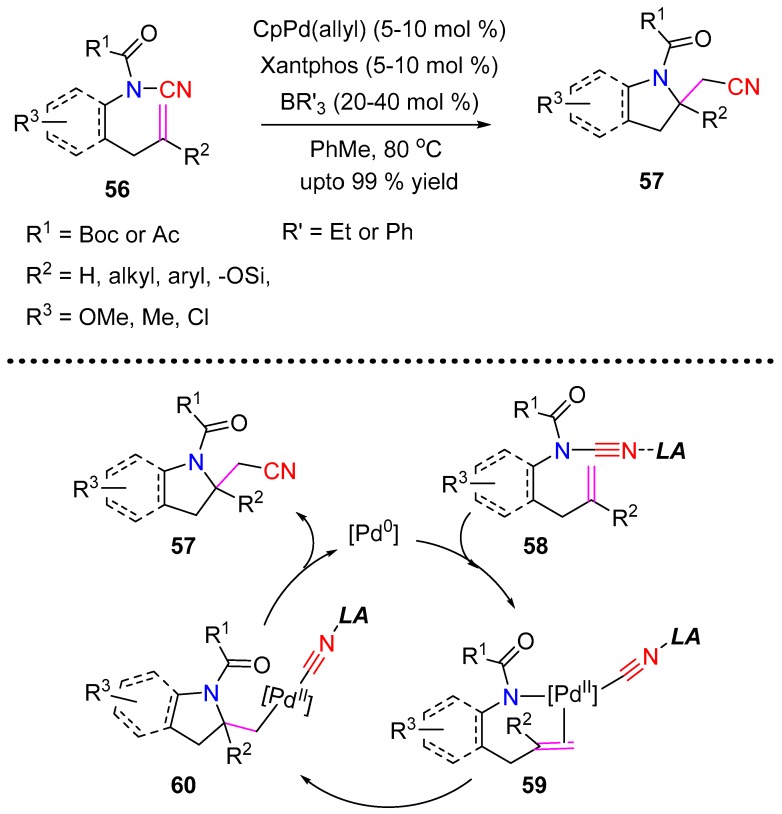
Palladium/triorganoboron cooperative catalysis for aminocyanation.

Metal catalyzed aminocyanation involves the simultaneous installment of an amino and nitrile-group on adjacent carbon atoms, commonly in unsaturated systems. Unlike its congeners, oxycyanation, carbocyanation, thiocyanation etc., the field of aminocyanation is relatively unexplored. The first example of aminocyanation via the cleavage of the N–CN bond by a cooperative palladium/triorganoboron catalysis was reported by Nakao et al. in 2014 [28]. An intramolecular insertion of N–CN into the double bond of *N*-cyano-*N*-[2-(2-methylallyl)aryl]acetamide **56** was achieved under the catalytic system: CpPd(allyl), 4,5-bis-(diphenylphosphino)-9,9-dimethylxanthene (xantphos), and BEt_3._ The 5-*exo*-trig products were obtained in good to excellent yield under elevated temperature (Scheme 14). The same authors also investigated the enantioselective aminocyanation, by replacing the xantphos ligand with an (*R,R,R*)-Ph-SKP ligand. A catalytic cycle was proposed, wherein the boron Lewis acid coordinated to the N–CN group **58** undergo oxidative addition to a Pd(0)−xantphos complex. *Syn-*aminopalladation of **59** in an *exo*-trig fashion followed by C−CN bond-forming reductive elimination generates the boron-bound product (Scheme 14).

**Scheme 15:**
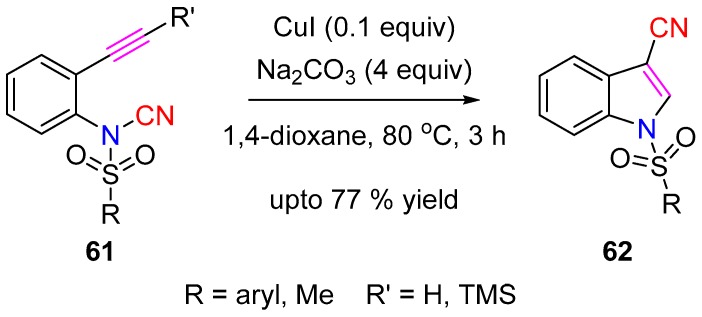
Copper catalyzed intramolecular aminocyanation.

Chien and Co-workers synthesized a series of 1-sulfonyl-3-cyanoindole (**62**) products by CuI catalyzed intramolecular aminocyanation of *N*-(2-ethynylphenyl)-*N*-sulfonyl-cyanamides **61** (Scheme 15) [29]. In the presence of CuI and Na_2_CO_3_, both terminal ethynyl and TMS-ethynyl-*N*-tosylcyanamide-substituted aryl-derivatives underwent intramolecular aminocyanation across the C–C triple bond leading to the 3-cyanoindole skeleton. Based on the experimental results with internal alkynes, it was postulated that the reaction proceeds via the formation of a key Cu-acetylide intermediate.

**Scheme 16:**
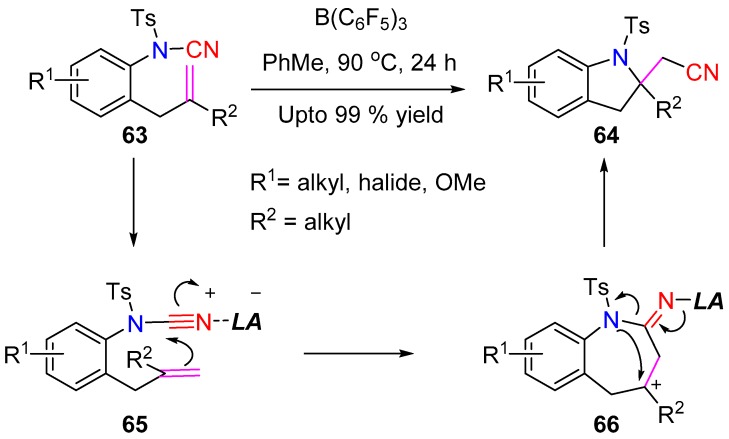
Lewis acid activated cleavage of N–CN bond for intramolecular aminocyanation.

A metal free intramolecular aminocyanation was achieved under Lewis acid catalysis by Douglas et al. which proceeds via cleavage of the N–CN bond of cyanamide [30]. The coordination of the Lewis acid catalyst with the sulfonyl-group lead to the weakening of the key N–CN bond, thereby inducing bond cleavage. Hence, starting with a variety of *N*-tosylcyanamides **63**, the indoline products **64** were obtained in good yields when treated with equivalent quantity of B(C_6_F_5_)_3_. The authors proposed a mechanism consistent with nucleophilic attack of the alkene on the nitrile center, which is supported by experimental data (Scheme 16). A comparable yield was obtained when the reaction was carried out with 20 mol % of Lewis acid, although extended reaction times were required. Initially the reaction was performed with a rhodium complex, but further investigation indicated that the N–CN bond activation can be achieved without the addition of any transition metal catalysts.

**Scheme 17:**
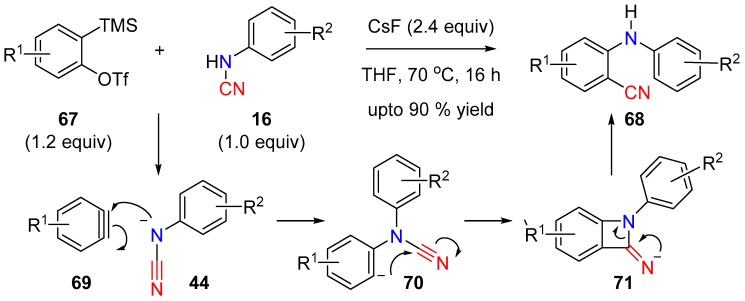
Aminocyanation of arynes.

Another example of a metal-free cleavage of the N–CN bond was described by Zeng et al. [31]. The researchers demonstrated that aryne-insertion across the N–CN bond to deliver the amino benzonitriles (**68**) could be achieved by in situ generation of arynes **69** and an anionic species **44** from the mono-substituted cyanamide. Both of the reactive species were generated simultaneously via a fluoride ion source (CsF). The aryne underwent nucleophilic addition to give the phenyl-anion **70**, which on cyclization gave the highly strained four-membered intermediate **71**. Ring opening of the intermediate **71** and protonation led to the final aminocyanated product (Scheme 17).

#### 3.2.2. Aminating Agent

**Scheme 18:**
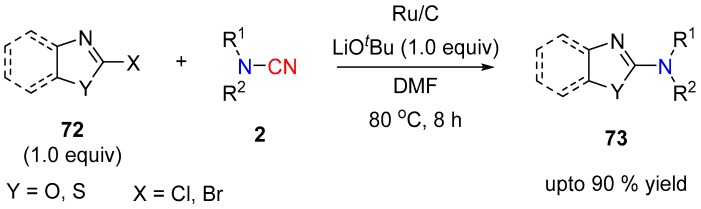
Cyanamide as aminating agent.

In 2014 Nageswar and co-workers reported the amination of 2-halobenzothiazole and benzoxazole derivatives on reaction with dialkyl cyanamides under Ru/C catalysis (Scheme 18). The reaction utilizes cyanamides as aminating agent with the concomitant loss of nitrile group [32].

#### 3.2.3. Electrophilic Cyanation

*N*-Sulfonyl cyanamides viz *N*-cyano-*N*-tosyl-sulfonamide (**74**, NCTS), also known as *N*-cyano-*N*-phenyl-*p*-methylbenzene-sulfonamide [33], are popular cyanamide derivatives with significant applications in synthetic chemistry. The reactivity and stereoelectronic properties of the sulfonyl group have been exploited to orchestrate diverse reactions of cyanamides; e.g., Lewis acid assisted aminocyanation [30], generation of cyanamide anion by action of a nucleophilic fluoride ion [19], electrophilic cyanation by reaction with Grignard reagents [34] as well as for heterocyclic ring construction [35,36]. Since the re-introduction of NCTS by Beller et al. in 2011 as an efficient electrophilic cyanation source of aryl- and alkenyl-substrates under rhodium catalysis [37], there has been a growing interest in developing new catalytic metal systems for such reactions [38,39].

**Scheme 19:**
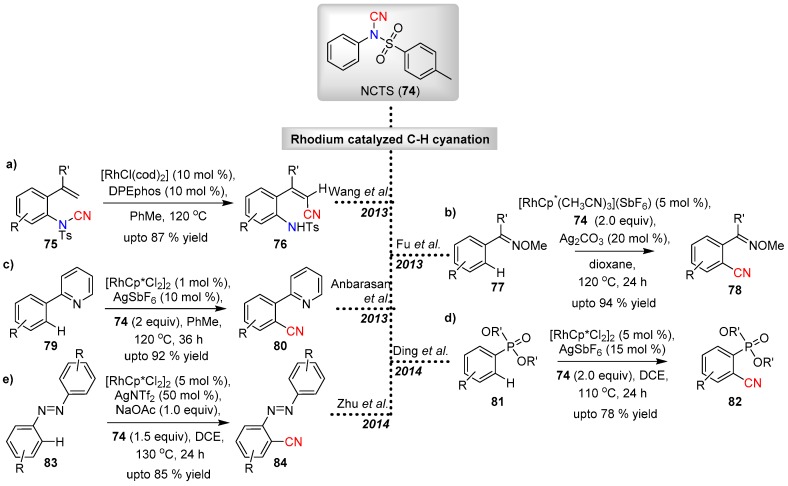
Rhodium-catalyzed electrophilic cyanation with NCTS.

Wang and co-workers reported the first example of rhodium-catalyzed intramolecular C–H cyanation in 2013 [40]. The intramolecular C–H cyanation of an appropriately decorated styrene substrate (**75**) was achieved via a rhodium(I)-catalyzed N–CN cleavage (Scheme 19a). Good substrate tolerance was shown for different substituents attached on the arene ring and styrene α-position. Alkyl-substituents in the olefinic α-position of the styrene has led to acrylonitriles in good yields, whereas corresponding arene substitution on the olefinic α-position, resulted in moderate yields of the cyanated products. The reaction establishes the importance of α-substitution in styrene for the cyanation to be feasible.

Fu and co-workers reported the syntheses of aromatic nitriles **78** under Rh-catalyzed directed C−H cyanation using NCTS as the cyanating agent (Scheme 19b) [41]. The cyanation was first developed for acetophenone *O*-methyl oxime substrates (**77**) and eventually extended to different directing groups such as pyridines, pyrazoles, dihydroimidazoles and dihydrooxazoles, which all resulted in moderate to good yields of the nitrile products. The reaction was found to tolerate a variety of synthetically demanding functional groups (e.g., unprotected phenol, Ar-I, epoxide), which proved to be challenging with other transition metal catalysts such as Pd.

A series of reports on the chelation assisted C–H activation for cyanation under Rh-catalysis with NCTS have since appeared. Anbarasan et al. described the cyanation of phenyl pyridines **79** with [RhCp*Cl_2_]_2_ catalyst in the presence of AgSbF_6_ (Scheme 19c) [42]. This method allowed the synthesis of various aryl nitrile derivatives in good to excellent yields, while reducing the catalytic loading to 1 mol %.

The H.-Y. Ding group on the other hand employed the chelation-assisted *ortho*-cyanation on arylphosphate substrates **81** under rhodium catalysis (Scheme 19d) [43]. The protocol delivered complete *ortho*-selectivity, thereby hindering the formation of biscyanated aryl-phosphonate byproducts.

Zhu and co-workers applied the rhodium based catalytic protocol for C–H cyanation of symmetric azobenzenes (**83**) at the *ortho*-position (Scheme 19e) [44]. The protocol was more effective for the cyanation of aromatic azo-compounds having electron donating groups than those with electron withdrawing groups.

**Scheme 20:**
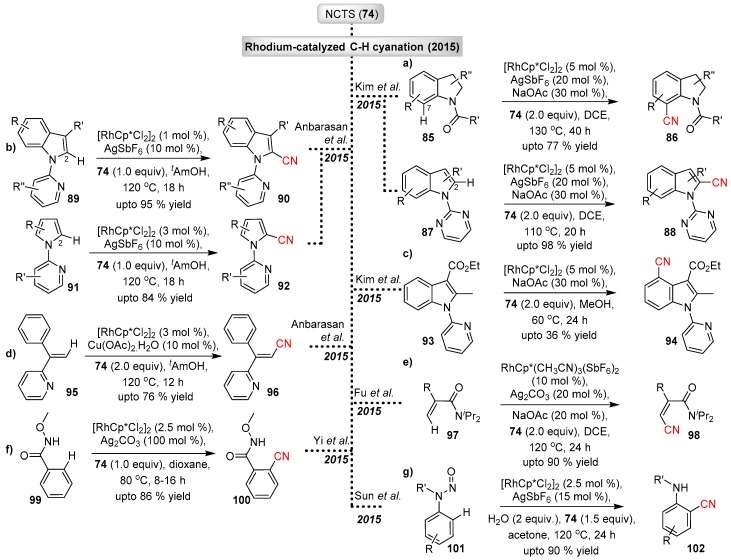
Rhodium catalyzed electrophilic cyanation with NCTS.

Chelation assisted C–H functionalization was extended to the indole and indoline scaffolds. Indole and indolines are ubiquitous structural motifs found in natural products and find application in pharmaceuticals, agrochemicals and dyes. The introduction of cyano groups to these structural motifs provides a means of decorating the indole and indoline rings. In particular, selective C-2 cyanation of indoles and C-7 cyanation of indolines have received much attention. In 2015, Kim et al. reported site selective, directing group-assisted C−H cyanation of indolines and indoles at the C-7 and C-2 positions respectively, via rhodium-catalyzed reaction with NCTS (Scheme 20a) [45]. The results indicated that *N*-carbonyl or carbamoyl-directing groups act very effectively in the C-7 cyanation (compounds **85** to **86**), whereas C-2, C-3 or C-4 substituted *N*-pivaloyl-indolines deliver the C-7 cyanated products only in moderate to good yields. A similar protocol was used in the synthesis of C2-cyanated indoles **88**, utilizing a removable pyrimidyl-directing group to achieve the desired product in excellent yields. Extending their work, Kim et al. reported the C-7 cyanation of C-2 substituted indoles **93** and **94** (Scheme 20c) [46].

Appearing at a similar time as Kim’s report, a publication by the Anbarasan group described the electrophilic C-2 selective cyanation of indoles and pyroles (Scheme 20b) [47]. The cyanation process was assisted by a removable directing group such as *N*-pyridyl, *N*-quinolyl and *N*-pyrimidyl. The 2-cyanoindoles and pyrrole products were obtained in the presence of low catalyst loadings (1–3 mol %) and additives.

Concurrently to the C–H cyanation study of indoles and pyrroles, Anbarasan and co-workers also reported the C–H cyanation of acrylonitriles/vinyl-nitriles (**95**) with NCTS via rhodium(III) catalysis (Scheme 20d). In this case, Cu(OAc)_2_ was used as an additive [48]. The new method tolerates a variety of functional groups, thereby allowing the synthesis of diverse substituted acrylonitriles **96**.

Rhodium catalyzed C–H cyanation of substituted vinyl amides **97** with NCTS to access alkenyl-nitriles (**98**) was accomplished by Fu et al. (Scheme 20e) [49]. This methodology exhibited good functional group tolerance and both acrylamides and ketoximes could be used as directing groups.

Expanding the scope of the rhodium catalyzed C−H cyanation with NCTS, Yi and co-workers reported the C−H cyanation of a wide range of *ortho*-substituted *N*-methoxybenzamides **99** (Scheme 20f). The reactions showed good substrate and functional group tolerance [50]. The new protocol delivers products with good regioselectivity and under mild reaction conditions.

Recently Sun et al. reported the synthesis of 2-(alkylamino)benzonitriles **102** via a rhodium catalyzed aryl C−H cyanation and denitrosation of *N*-nitrosoarylamines **101** with NCTS (Scheme 20g) [51]. The new reaction demonstrated good tolerance to a wide range of substituents on the aryl-ring and amino-group, while delivering the corresponding products in moderate to good yields.

In addition to rhodium, cobalt and ruthenium catalysts have also been developed as efficient transition metal catalysts for the electrophilic cyanation of C–H bond by NCTS.

**Scheme 21:**
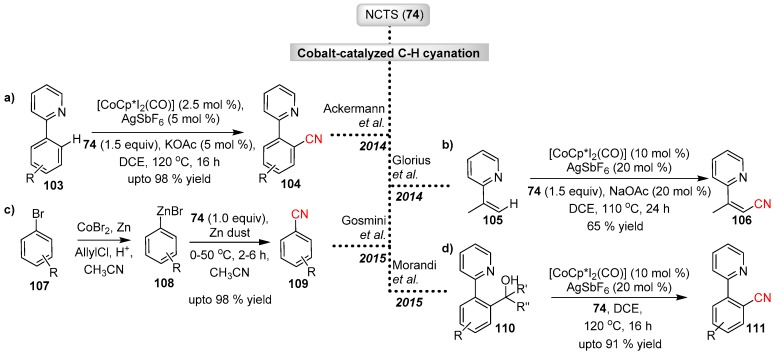
Cobalt catalyzed electrophilic cyanation with NCTS.

The first example of a cobalt-catalyzed cyanation of C–H bonds with NCTS was reported by Ackermann and Li in 2014 (Scheme 21a) [52]. The reaction was found to be dependent on the co-catalyst AgSbF_6_ and carboxylate additives. Mechanistic studies indicate that in situ generation of the highly active cationic cobalt(III) acetate is vital for site-selective *ortho*-cyanation. The methodology is also applicable to removable directing groups such as pyridyl, pyrimidyl and pyrazole.

In 2014 Glorius and co-workers reported the C–H cyanation of heteroaryls (*ortho*-cyanation), indoles (C-2 cyanation), and olefins under cobalt catalysis (Scheme 21b) [53]. The method exhibited good functional group tolerance and high efficacy when used in combination with different directing groups (pyridyl, pyrimidyl and pyrazole).

Recently the Gosmini’s group reported the C–H cyanation of aryl-zinc compounds catalyzed by cobalt(II) [54]. The study was inspired by utilization of mild reaction conditions and one-pot synthesis of aryl-zinc derivatives from aryl-halides and subsequent cyanation using the same cobalt(II) catalyst (Scheme 21c). In contrast to previous reports, Gosmini’s protocol uses low valent cobalt(II) catalysts. However, in this case, a catalytic amount of zinc dust was used to release the reactive cobalt species in the cyanation step. Despite the high tolerance level, the authors report that the protocol is ineffective in the presence of a strong chelating group (ketone or nitrile) on the aryl-zinc species.

Recently Morandi et al. described the first cobalt(III) catalyzed C–C bond cleavage of secondary and tertiary alcohols and subsequent electrophilic cyanation by NCTS (Scheme 21d) [55]. In this protocol, directing groups such as pyridyl are important to both facilitate the β-carbon elimination and stabilize the cobalt-aryl intermediate against proto-demetallation prior to electrophile/[CN]^+^ trapping. Mechanistic studies indicate that the cyanation takes place directly from the cobalt-aryl intermediate rather than prior protonation followed by C–H activation.

**Scheme 22:**
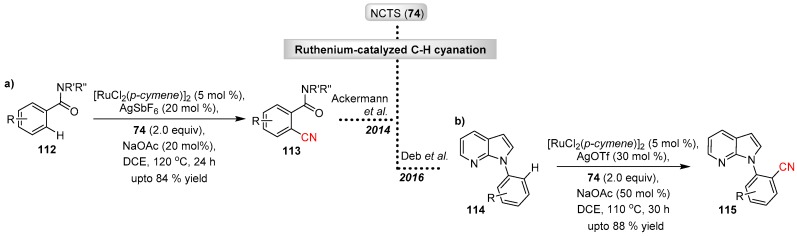
Ruthenium catalyzed electrophilic cyanation with NCTS.

The Ackermann group have also explored the applicability of ruthenium(II) catalysts for C–H cyanation with NCTS (Scheme 22a) [56]. Ackermann explored the feasibility of a ruthenium catalyst with weakly coordinating amide groups for chelation assisted directed C–H cyanation of arenes and heteroarenes. The protocol proved to be effective, demonstrating good substrate scope and tolerance to a wide range of functional groups.

Deb et al. demonstrated ruthenium(II) catalyzed *ortho*-aryl C–H cyanation of azoindoles with NCTS (Scheme 22b) [57].

**Scheme 23:**
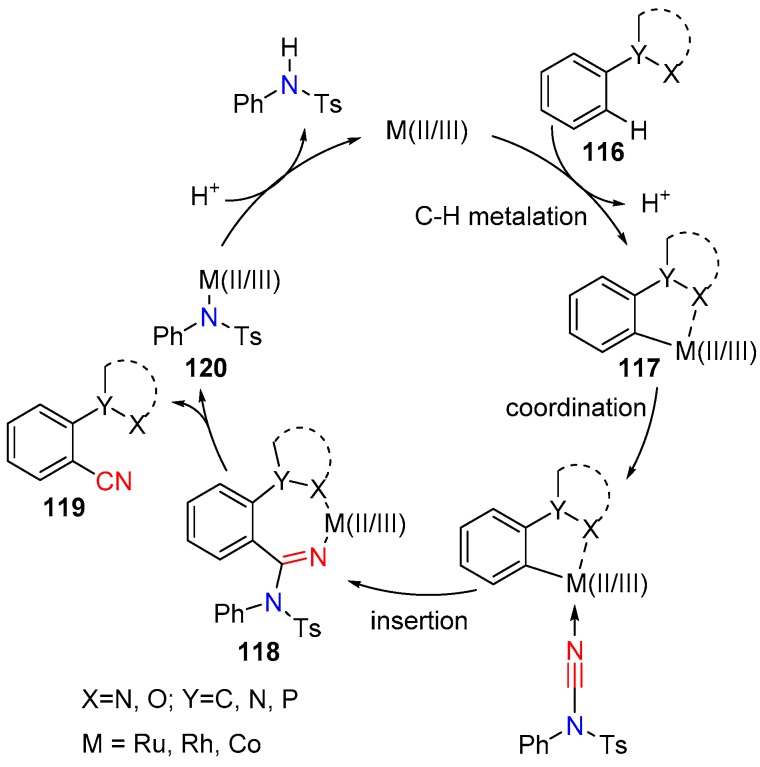
General catalytic cycle for metal catalyzed [Ru, Rh, Co] C–H activation and cyanation.

A general mechanism can be proposed for the metal catalyzed reactions: Initial C–H metalation is followed by the coordination and migratory insertion of NCTS. Finally, β-elimination and proto-demetallation results in the corresponding product and the regenerated cationic M(II/III) catalyst (Scheme 23).

**Scheme 24:**
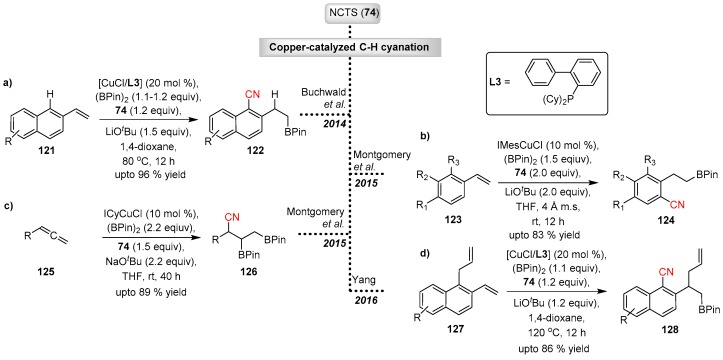
Copper-catalyzed electrophilic cyanation with NCTS.

**Scheme 25:**
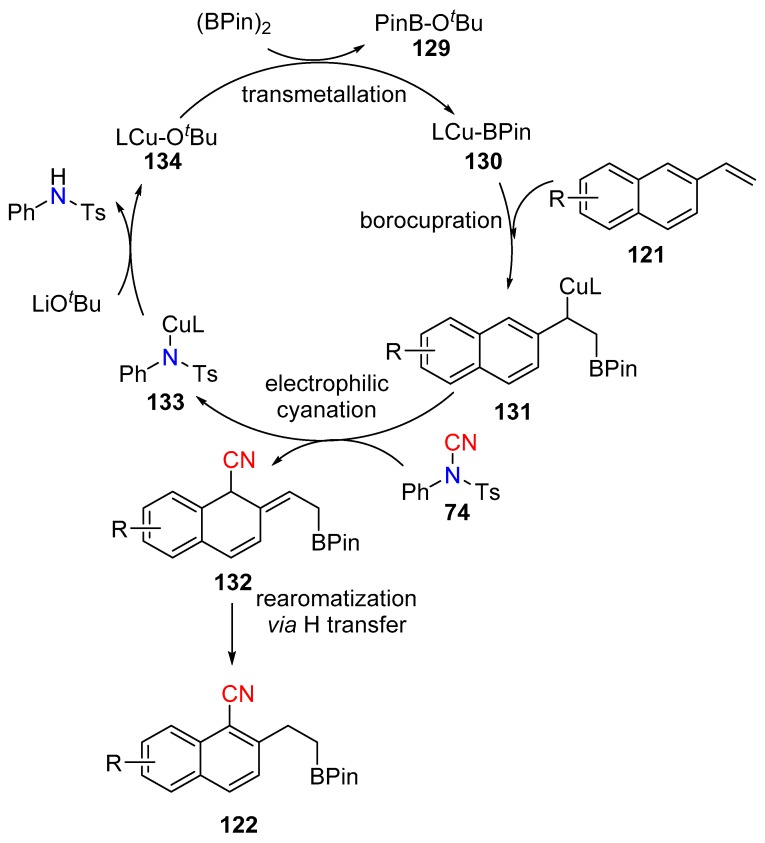
Copper catalytic cycle for C–H cyanation.

In 2014 Buchwald and Yang reported the copper catalyzed *ortho* C−H cyanation of vinylarenes (**121**) with NCTS (Scheme 24a) [58]. Inspired by using π-directing groups instead of strong σ-directing groups, the team developed the reaction with vinyl-arenes. Here, the C–C double bond acts as both a reaction site, which undergoes simultaneous borylation, and as a directing group. The proposed mechanism involves transmetallation of the phosphine-ligated copper catalyst with the diboron reagent, which then undergoes subsequent borocupration to form the benzyl-copper intermediate **131**. Electrophilic *ortho*-cyanation takes place on the benzyl-copper intermediate with NCTS to give the dearomatized cyanation product **132**, which transforms to the aromatized product **122** via rapid H-transfer (Scheme 25). The mechanism was further explored by Yang and Liu. Diverse vinyl naphthylenes bearing both electron donating and withdrawing groups undergo this site-selective transformation in good yield. The resulting borylated compounds can be diversified effectively into other useful complex molecules.

Exploring the simultaneous *ortho*-cyanation and hydroboration of styrene derivatives with NCTS/(BPin)_2_ system, Montgomery et al. disclosed the use of *N*-heterocyclic carbene (NHC)-ligated Cu-catalysts (Scheme 24b) [59]. The protocol required mild reaction conditions and was compatible with a broad range of substituted styrenes, resulting in selective *ortho*-cyanation on the least substituted *ortho*-position. Polycyclic substrates with extended conjugation gave the cyanation on the adjacent ring structure. The resulting products were further derivatized to indanone derivatives via a novel AgNO_3_/Selectfluor-mediated radical cyclization. This two-step protocol provides an efficient method for cyclopentannulation of styrenes.

The group also reported an unprecedented copper-NHC based catalyst system for the cyanation and diborylation of terminal allenes **125** with NCTS/(BPin)_2_ (Scheme 24c). The protocol exhibited exceptional chemo-, region- and diastereoslectivity, while being compatible with trifunctionalization of a variety of terminal allenes [60].

More recently, Yang applied NCTS for cyanation of 1-allyl-2-vinylnaphthalenes **127** under copper catalysis (Scheme 24d) [61]. Similar to previous work, this reaction cascade was initiated by a copper catalyzed electrophilic dearomatization process. The resulting dearomatized intermediate undergoes a regio and stereospecific 1,3-transposition of an allyl-fragment via a rearomatizing Cope rearrangement to give the final compound **128**. This method also demonstrated promising results against a wide range of substrates differing at the naphthyl moiety and the migrating allyl fragment.

**Scheme 26:**
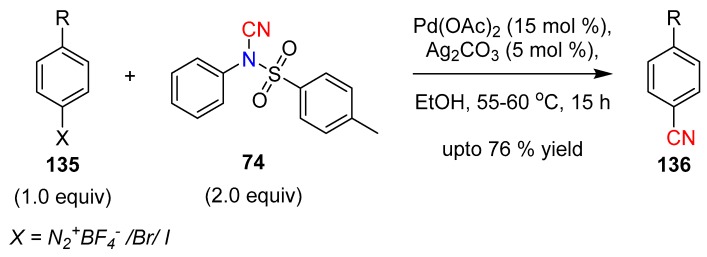
Palladium-catalyzed electrophilic cyanation with NCTS.

Cyanation of arene diazonium salts are known to be challenging due to homo-coupling at high temperatures and ready decomposition. Lee et al. reported a palladium based electrophilic cyanation protocol for synthetically challenging arenediazonium tetrafluoroborates and aryl halides with NCTS under mild reaction conditions [62]. The authors emphasized the importance of using a polar solvent (e.g., EtOH) for the reaction, presumably due to its involvement in the reduction of Pd(II) to Pd(0) together with the generation of CH_3_COOH (Scheme 26).

While NCTS is the reagent of choice for many transition metal catalyzed cyanation protocols, efforts have also been directed towards the development of transition-metal-free conditions. An early example of NCTS for cyanation under metal-free conditions was reported by the groups of Beller and Wang [4,63]. Beller and colleagues employed NCTS in the direct cyanation of aryl and heteroaryl-halides via in situ generated Grignard reagents, to synthesize a diverse array of benzonitrile derivatives. Meanwhile the Wang group demonstrated the applicability of NCTS for the site-selective cyanation of indoles and pyrroles under Lewis acid catalysis.

**Scheme 27:**
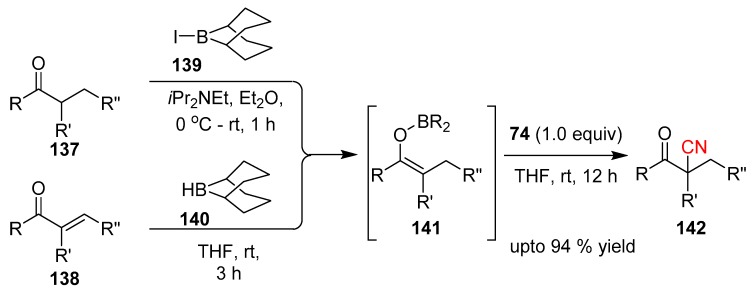
Metal free cyanation of boronenolates.

In 2016 Minakata et al. reported the cyanation of boron enolates **141** with NCTS [64]. Inspired by synthesizing β-ketonitriles **142**, which are important building blocks in the synthesis of different heterocycles (pyrazoles, pyrimidines, thiophenes), Minakata planned to intercept the electrophilic nitrile (NCTS) with an enolate. Boron enolates were derived from either the ketone **137** or the α,β-unsaturated ketone **138** and the resulting enolate **141** was reacted with NCTS under milder reaction conditions. Even though the mechanistic aspect of the reaction is yet not fully resolved, the pathway is believed to be initiated by the activation of NCTS via coordination to the Lewis acidic boron center. This coordination is also assumed to enhance the nucleophilicity of the boron-enolate, promoting the cyanation. A diverse range of β-ketonitriles were synthesized through this protocol exhibiting ample scope for substrates (Scheme 27).

### 3.3. Cyanamides in Radical Reactions

Cyanamide-based radical cascade reactions are emerging as an important tool to access nitrogen-containing polycyclic frameworks. These compounds are key intermediates in heterocyclic natural products. The radical cascade reactions allow rapid access to complex cores, initiated with simple starting materials, while maintaining not only atom- and step-economy, but also high stereo-selectivity. The seminal work in this area was reported by Fensterbank et al [65,66].

**Scheme 28:**
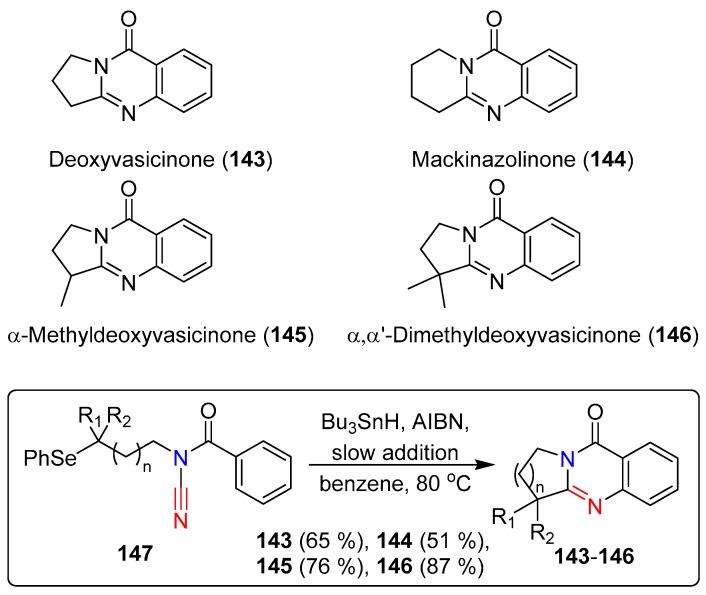
Radical reaction of *N*-acyl-cyanamides for the synthesis of guanidine based natural products.

**Scheme 29:**
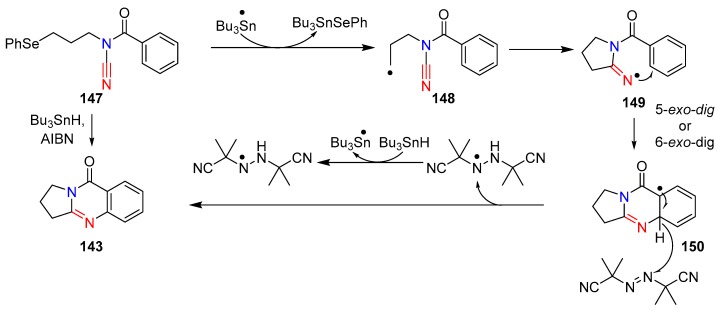
Proposed mechanism for the radical cascade involving *N*-acyl-cyanamide to generate guanidine derivatives.

Extending their previously reported radical cascade reactions of *N*-acyl-cyanamides to generate guanidine derivatives, Fensterbank et al. achieved the syntheses of two natural products; deoxyvasicinone (**143**) and mackinazolinone (**144**). The group also synthesized two guanidine analogues, α-methyldeoxyvasicinone (**145**) and α,α’-dimethyldeoxyvasicinone (**146**) [67]. The 5,6,6-and 6,6,6-tricyclic guanidine derivatives were successfully synthesized in good yields by reacting *N*-acylcyanamide with Bu_3_SnH and AIBN in benzene under reflux conditions (Scheme 28). This protocol was not only effective with the previously reported aminyl- and vinyl-radicals, but also compatible with alkyl-radicals. Alkyl substituted *N*-acylcyanamides were cyclized in both 5- and 6-*exo*-dig pathways, although the 6-*exo*-dig cyclization was comparatively slower. Attempts to achieve tin-free reaction conditions with (TMS)_3_SiH resulted in substantially lower yields. The mechanism of the reaction was postulated to proceed with the formation of an alkyl radical via the abstraction of phenyl-selenide by a tributyltin-radical. The resulting alkyl-radical undergoes 5- or 6-*exo*-dig cyclization, forming the amide-iminyl-radical, which itself undergoes a final rearomatization–cyclization through β-hydrogen abstraction by AIBN (Scheme 29).

**Scheme 30:**
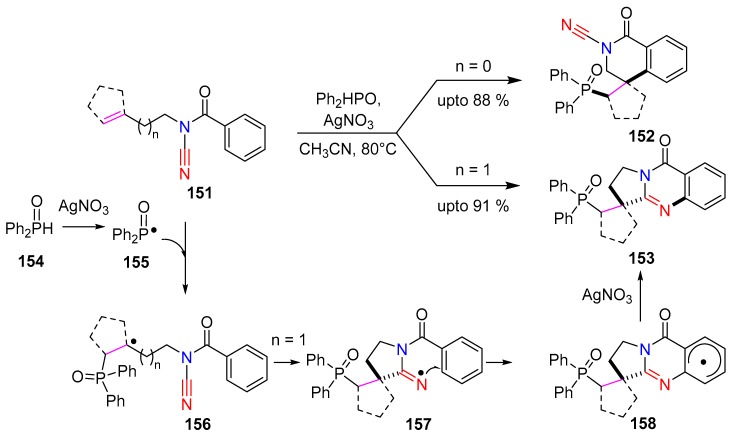
Phosphorylation/cyclization radical cascade of *N*-acyl cyanamide alkenes.

Cui and co-workers applied a radical cascade cyclization protocol to construct dihydroisoquinolinone and 4-quinazolinone cores **152** and **153**. The methodology involved a silver(I) mediated phosphorylation/cyclization radical cascade of *N*-acyl-cyanamide alkenes [68]. A wide range of phosphorous quinazolinone derivatives were achieved in good yields by reacting *N*-acyl-cyanamide alkenes **151** (1 equiv.) and diphenylphosphine oxide (**154**, 2 equiv.) in the presence of AgNO_3_ (1 equiv.) in CH_3_CN. Following this protocol, the *para*-substituted *N*-acylcyanamidebutenes delivered phosphorous 4-quinolinones in moderate to excellent yields, while exhibiting a broad functional group compatibility (Scheme 30). When *meta*-substituted cyanamides were subjected to the reaction, a mixture of regioisomeric phosphorous 4-quinolinones resulted. The protocol was also amenable for heterocyclic moieties such as indoles and pyrazoles. Di-substituted alkenes were more effective compared to mono-substituted alkenes, which can be attributed to the stabilization of the in situ generated alkyl-radical intermediate.

Interestingly, the reaction of unactivated cyclic internal alkenes resulted in a range of spirocyclic compounds. Dihydroquinolinones were obtained in moderate to good yields when *N*-acylcyanamide-propenes was subjected to cyclization protocol. The feasibility of both phosphonates and phosphine oxides substrates were investigated with positive outcomes. The phosphonate addition was particularly attractive as it gave access to the phosphonic acid derivatives. The authors reported that the addition of a radical scavenger resulted in the quenching of the reaction, which is indicative of a radical pathway being involved. The reaction is initiated with the formation of a diphenyl-phosphine-oxide-radical **155** and its subsequent trapping by the olefin to form the alkyl radical **156**. This undergoes a subsequent cascade reaction with the cyanamide nitrile to offer the final cyclized product **153** (Scheme 30).

**Scheme 31:**
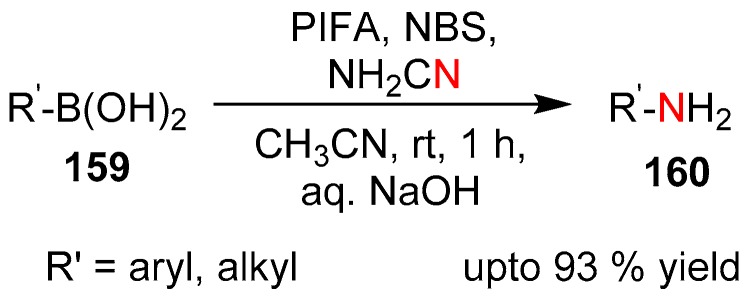
Synthesis of primary amines by reaction of cyanamide-radical with boronic acids.

Goswami et al. disclosed a metal and base free protocol for chemo-selective generation of primary amines via *ipso*-amination of substituted boronic acids using cyanamide radicals as the aminating agent [69]. They envisaged that radical transfer to the aryl cyanamides occurred via succinimidyl-radicals, which itself was generated in situ by the action of a hypervalent iodine derivatives such as phenyliodine-bis(trifluoroacetate) (PIFA) (Scheme 31).

Subsequently the cyanamide-radicals reacted with boronic acid derivatives via the nitrile nitrogen atom to give the primary amine products. The protocol was highly effective with both aliphatic and aromatic boronic acids and could tolerate a variety of functional groups. The likelihood of a radical pathway was confirmed by the addition of radical scavengers to the reaction medium.

### 3.4. Coordination Chemistry of Cyanamides

The organometallic complexes of cyanamide find application in materials sciences and more recently in the biological sciences [70,71]. Substituted cyanamides offer multiple coordination sites: (i) end on σ-bonding via the nitrile nitrogen (ii) side on π-bond via the nitrile group (iii) via the amine nitrogen and (iv) via the aromatic π-system in the aryl-substituted cyanamides. The coordination mode and strength of cyanamide bonding depends hugely on their steric and electronic properties; i.e., the number and kind of *N*-substitution; for example the monoalkyl- and dialkylcyanamides have increased electron density on the NCN system, whereas the aryl-substituted cyanamides offer extended π-conjugation into the aromatic ring. This provides an energetically favourable means through which a metal ion can couple into a conjugated organic π-system.

In addition, the mono-substituted cyanamides, such as phenylcyanamide (PhNHCN) are known to participate as coordinating ligands in neutral as well as anionic cyanamido form ([PNCN]^−^) [72].

The coordination chemistry of alkylcyanamide, arylcyanamide and dicyanamides has been previously covered in a number of excellent reviews [73,74]. The present section will provide an overview on the application of substituted cyanamide in coordination chemistry via a few selected representative examples from the recent literature. A more detailed discussion is beyond the scope of this article.

#### 3.4.1. Dialkyl-Cyanamide Complexes

**Scheme 32:**
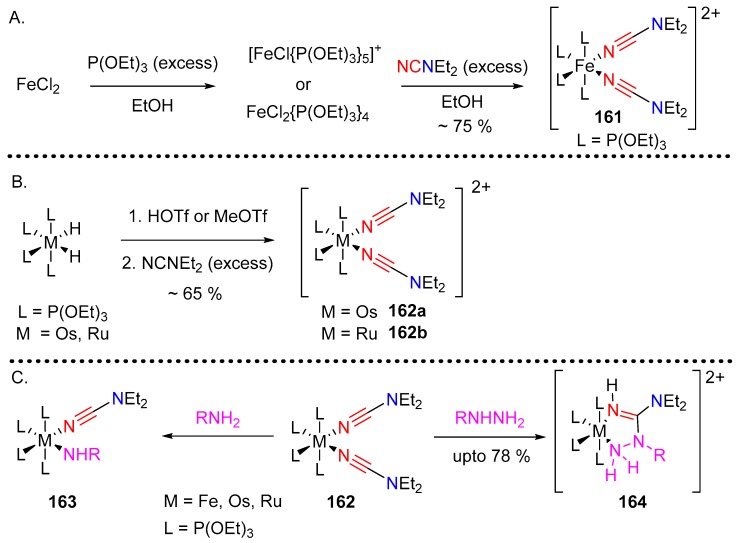
Reaction of amines with cyanamide metal complex.

Albertin et al. initiated a program to study the behavior of amino substituted nitriles, such as cyanamides and cyanaoguanidines, and their coordination chemistry with metals like iron, ruthenium and osmium [75,76]. The bivalent metallic bis-cyanamide complex of iron **161** was obtained by mixing FeCl_2_ with P(OEt)_3_ and then treating with dialkyl-cyanamide followed by addition of an excess of NaBPh_4_ (Scheme 32A). The corresponding osmium and ruthenium-derived complexes **162a** and **162b** were obtained by treating the corresponding metal hydride (MH_2_L_4_) with one equivalent of HOTf or MeOTf followed by addition of excess of dialkylcyanamide (Scheme 32B). The authors further studied the reaction of these complexes with nucleophilic hydrazine. The reactions, in each case, produced a solid product characterized as hydrazinecarboximidamide complex **164**. The reaction was proposed to proceed via the displacement of a cyanamide ligand with hydrazine followed by intramolecular nucleophilic attack on the neighboring nitrile carbon by the second amino group of hydrazine leading to the five membered azametallocycle (Scheme 32C).

In comparison the reaction of the iron triad with aliphatic amines like *^i^*PrNH_2_ and *^n^*PrNH_2_ led to the mono-substituted mixed ligand product only with no observed nucleophilic attack on the cyanamide nitrile. No mixed ligand product was observed upon reaction with the corresponding aromatic amine and NH_3_.

Recently Bokach et al. reported the synthesis of two Co(II) (dimethylcyanamide) complexes along with the structural details of their dihydrate complexes *trans*-[Co(H_2_O)_4_(NCNMe_2_)_2_]X_2_·2H_2_O [77]. A key feature of the X-ray crystal structure was the hydrogen bonding of the counter anion and the ligated water of crystallization. They studied the effect of both chloride and bromide counter ions and associated water molecules on the 3D network of hydrogen bonding, and rationalized the differences observed based on the electronic differences primarily between the counter ions in terms of electrostatics but also depending on the metal center size and electronegativity.

#### 3.4.2. Aryl Cyanamide Complexes

The electronic properties of substituted cyanamides are known to play a crucial role as a bridging ligand for metal-metal coupling in dinuclear complexes, which directly affects their magnetic properties [78]. In 2013 Crutchley et al. synthesized a series of nine mononuclear [Ru(Tp)(dppe)L] complexes, (where L is a substituted phenylcyanamide ligand, Tp is hydrotris(pyrazol-1-yl)-borate, dppe is 1,2-bis[(diphenyl-phosphino)ethane) and a dinuclear complex [{Ru(trpy)(bpy)}_2_(μ-adpc)]^2+^, (where trpy is 2,2′:6′,2″-terpyridine, and bpy is 2,2′-bipyridine and adpc^2−^ is azo-4,4′-diphenylcyanamide), of ruthenium with substituted phenylcyanamide ligands [79]. The crystal structure of [Ru(Tp)(dppe) (Cl_5_pcyd)] and [{Ru(Tp)(dppe)}_2_(μ-adpc)] revealed that the cyanamide coordinates to the ruthenium(II) through the terminal nitrogen. Significant conjugation between the cyanamide and phenyl ring was predicted, given the planarity of the cyanamide group in the complex.

A mixed valence complex was observed for these complexes upon oxidation, which showed strong π-interactions between Ru(III) and the cyanamide group, operating through the super exchange mechanism. The authors also investigated the effect of disrupting the strong π-interactions through a scorpionate ligand, which possesses strong π-donor properties via the anionic coordination mode and weakens the inter-metal coupling. This allowed an overall charge reduction, whilst retaining the properties of the original mixed-valence complex.

The Chiniforoshan group have been investigating coordination complexes of a range of transition metal with cyanamide derivative ligands and their possible medicinal activity. The group recently reported the synthesis and characterization of Ni(II) and Cd(II) complexes with 4-nitrophenylcyanamido ligands [80,81]. More recently the group reported the synthesis and biological activities of the polymeric coordination complexes of mercury(II), with the anions of a number of arylcyanamides [82]. The complexes were obtained via deprotonation of the relevant arylcyanamide with NaOH, followed by mixing the arylcyanamide anion with Hg(NO_3_)_2_·H_2_O in acetone at reflux temperature.

**Figure 1:**
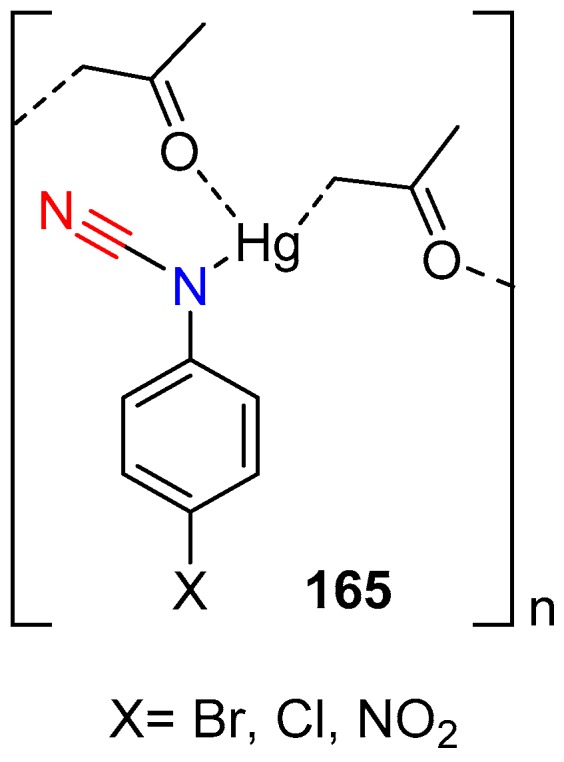
A schematic representation of Mercury based polymeric coordination complex.

The structural analysis of the solid products revealed that the cyanamide ligand is coordinated through the amine nitrogen, which is relatively rare. The nitrile coordination mode was observed, but was thermodynamically less favorable than the amine isomer. Analysis also revealed that the anion of the acetone, the reaction solvent, was present as a bridging ligand connecting two mercury ions through the carbon atom of one and carbonyl oxygen (C=O) atom of another acetone anion. Antimicrobial assays indicated some inhibition to bacterial growth, but there was no overall improvement when compared the free ligand itself (Figure 1).

**Figure 2:**
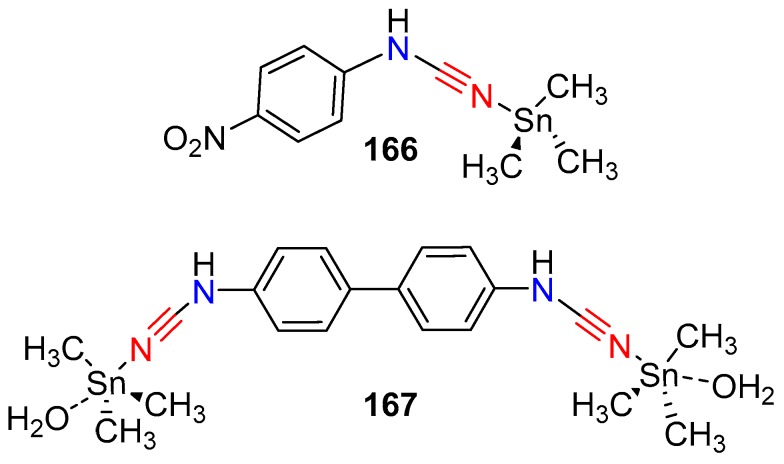
Structure of trimethyltin derivatives.

The group also reported the synthesis of trimethyltin(IV) complexes with 4-nitrophenylcyanamide and a biphenyl derivative of cyanamide ligand [83]. The complexes were obtained by mixing the trialkyl-tin with the ligand in an alkali medium (NaOH), and irradiating the mixture under high-intensity ultrasound. In these complexes the cyanamide coordinates to the metal through the nitrile nitrogen; the biphenyl derivative was found to bridge between two trimethyltin metal centers (Figure 2). All compounds showed some activity in the antimicrobial/antifungal assay, but did not exceed the efficacy of ciprofloxacin or fluconazole for antimicrobial or antifungal activity. However, the cytotoxic activity of these compounds were impressive against the HeLa cell line. Both complexes had similar efficacy in causing cell death in the MTT assay, and this was comparable to *cis*-platin.

**Figure 3:**
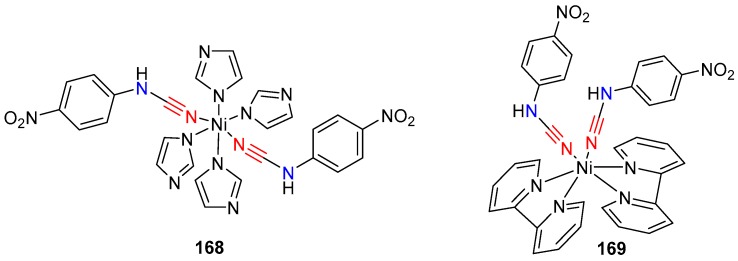
Representative drawing of nickel cyanamide complexes.

Recently, Chiniforoshan et al. synthesized three mononuclear complexes of nickel(II) with 4-nitrophenyl-cyanamide and their antimicrobial and cytotoxic properties were investigated [71]. The three complexes [Ni(HIm)_4_(4-NO_2_pcyd)_2_]·CH_3_OH, [Ni(bpy)_2_(4-NO_2_pcyd)_2_]·CH_3_CH_2_OH, and [Ni(phen)_2_(4-NO_2_pcyd)_2_]·CH_3_OH were readily obtained by the reaction of 4-nitrophenylcyanamide ligand with Ni(OAc)_2_·4H_2_O in the presence of 1,10-phenanthroline (phen), 2,20-bipyridine (bpy), imidazole (HIm) respectively. X-ray crystallographic studies of the complexes **168** and **169** revealed that in each case, the cyanamide ligand is coordinated via the terminal nitrile nitrogen rather than the sterically hindered amine nitrogen (Figure 3). The observed bond angles and bond distances between the metal-ligand and those of N–C–N of the cyanamide ligand suggested that the coordination mode of the cyanamide group to Ni(II) ion is approximately linear. This finding helps to confirm the extended conjugation of the cyanamide π-electrons into the aromatic ring and thereby the ability of the Ni(II) to accept π-density into a filled d_π_ orbital. The authors further conducted biological studies and tested the cytotoxicity and antimicrobial potential of these complexes.

Efficacy was shown by all of the complexes against human lung adenocarcinoma (A549), prostate (Du145), epithelial carcinoma (HeLa) and breast cancer (MCF-7) cell lines. When compared to *cis*-platin, higher or equal efficacy was shown by all compounds against Du145 and HeLa cell lines. This suggests that the compounds may be more effective against prostate and cervical cancers respectively. The compounds also had some antibacterial and antifungal activities.

## 4. Conclusions

Since the early reported use of cyanamides, the application of this unique functional moiety have diversified hugely, further enriching the field of amino-substituted nitrile chemistry. Here we have presented a selection of recent synthetic methodologies developed to access this special group of compounds, along with selected examples of the diverse synthetic applications. The number of reports in the recent years on substituted cyanamide aptly demonstrate the interest that this moiety has generated among synthetic chemists world-over. These new advances in cyanamide chemistry will no doubt attract further studies, thereby widening the applications in the relatively unchartered areas at the interface of chemistry, biology and material science.

## Supplementary Files

Supplementary File 1
