# Supplementary material for: Recent Advances in Cyanamide Chemistry: Synthesis and Applications
Source: Molecules. 2017 Apr 12;22(4):615. doi: 10.3390/molecules22040615 (PMC6154562; doi:10.3390/molecules22040615)
Supplement: Supplementary File 1 [file molecules-22-00615-s001.pdf]

Summary Table 1: *Summary of reactions discussed in the article and corresponding citations.*

Entry	***Reagents and Conditions***	***Ref. No.***
	**2. *Synthesis***	
	**2.1. *N*-Cyanation of secondary amine**	
	**2.1.1. Using electrophilic nitrile [CN]$^+$ reagents**	
1.	NaOCl (aq) (1.5 equiv), TMSCN (1.2 equiv), MeCN, 4 h, 23 °C	[5]
2.	2-Thiocyanoimidazolium salt (1.2 equiv), DIPEA, DCM, RT	[6]
3.	i) Cl_3CCN (1.1 equiv), CH_3CN, 23 h, RT; ii) NaO^tAm (2.0 equiv), DME, 0.5 h, RT	[7]
	**2.1.2 Under copper catalysis**	
4.	CuCN (2.0 equiv), Na_2SO_4 (2.0 equiv), $CuBr_2$ (5 mol %), TMEDA (2.0 equiv), CH_3CN, 4 h, RT	[8]
5.	AIBN (1.5 equiv),CuI (20 mol %), K_2CO_3 (2.0 equiv), CH_3CN, O_2, 75 °C	[9]
	**2.2 Cyanamides from amidoximes and guanidoximes**	
6.	Fe-Porphyrin Catalyst (10 mol %), H_2O_2 (2.0 equiv), [Bmim][PF_6]; 5h, RT	[10]
7.	A: *p*-TsCl (1.05 equiv), DIPEA (1.05 equiv), CH_2Cl_2 (0.1M); 3 h, 0 °C-RT<br>B: *o*-NsCl (1.2 equiv), DIPEA (1.2 equiv), CH_2Cl_2 (0.1M), 1 h, reflux<br>C: $ArSO_2Cl$ (1.05 equiv), Pyridine (1M); 0.5-12 h, 0 °C-RT	[12]
	**2.3 Cyanamides from isoselenocyanates**	
8.	i) NH_3 (1.2 equiv), DCM, RT; ii) [dibmim][BF_4] (1.0 equiv), THF, 0.5 h, RT	[13]
9.	NaN_3 (1.2 equiv), $PhCH_2Br$ (0.7 equiv), 1,4 Dioxane/H_2O (5 %)	[13]
	**3. *Synthetic Applications of Substituted Cyanamides***	
	**3.1.1. [3+2] Cycloaddition**	
11.	*With alkyne:* cyanamide (5.0 equiv), 2-picoline oxide (2.0 equiv), $Ph_3PAuNTf_2$ (3 mol %), PhCl, $MeSO_3H$, 60 °C, 2 h	[15]
12.	*With N-Boc-hydroxylamine:* i) cyanamide (1.0 equiv), $ZnCl_2$ (2.2 equiv), THF; 16h 20°C; ii) TFA/TFAA (5/1.2 equiv), DCM, 2h, 20°C.	[16]
13.	*With chloroxime :* p-tosyl cyanamide (1.2 equiv), TBAF (3.0 equiv, 3h addition) THF, 0°C	[17]
	**3.1.2 [2+2+2] Cycloaddition**	

14.	*With dialkyne derivative:* [Ni(cod)$_2$] (5 mol %), IMes (10 mol %), PhMe, 0.5 h, RT	[19]
15.	*With dialkyne derivative:* $FeCl_2$ (5 mol %), Zn (10 mol %) Ligand 1 (10 mol %), PhMe, 0.5 h, 70°C<br>*With terminal alkyne*: $FeCl_2$ (5 mol %), Zn (10 mol %), Ligand 1 (10 mol %), C_6H_6, 6 h, 50 °C	[20]
16.	*With alkenylnitrile derivative*: FeI_2 (5 mol %), Zn (30 mol %), Ligand 2 (10 mol %), PhMe, 12-72 h, 40 °C	[21]
17.	*With dialkyne derivative*: [Ir(cod)Cl]$_2$ (1 mol %), DPPF (2 mol %), C_6H_6, 1 h, reflux	[22]
	**3.2 N-CN bond cleavage**	
	**3.2.1 Aminocyanation**	
	**3.2.1.1 Metal catalysed**	
18.	*N-Acyl cyanamide derivative*: CpPd(allyl) (5-10 mol%), Xantphos (5-10 mol%, BEt_3 or BPh_3 (20-40 mol %), PhMe, 2-48 h, 80 °C	[25]
19.	*p-Tosyl cyanamide derivative*: CuI (0.1 equiv), Na_2CO_3 (4 equiv), 1,4-dioxane, 3 h, 80 °C	[26]
	**3.1.1.2 Metal Free**	
20.	*Intramolecular alkene aminocyanation*: $B(C_6F_5)_3$, PhMe, 24 h, 90 °C	[27]
21.	*Intermolecular benzyne aminocyanation*: phenylcyanamide (1.0 equiv), (CsF (2.4equiv), THF, 16 h, 70 °C	[28]
	**3.2.2 Aminating Agent**	
22.	*With benzisoxazole derivative and dialkylcyanamide:* Ru/C, LiO^{t}Bu (1.0 eq), DMF, 8 h, 80 °C	[29]
	**3.2.3 Electrophillic cyanation**	
	**3.2.3.1 Metal catalyzed**	
	**3.2.3.1.1 Cyanation under rhodium catalysis**	
23.	*Tosyl-cyanamide derivative*: [RhCl(cod)$_2$] (10 mol %), DPEphos (10 mol %), PhMe, 12-48 h, 120 °C	[35]
24.	*Methyl oxime derivative*: [RhCp(CH_3CN)$_3$](SbF_6) (5 mol %), NCTS (2.0 equiv), Ag_2CO_3 (20 mol %), 1,4-dioxane, 24 h, 120 °C.	[36]
25.	*Phenylpyridine derivative*: [Cp*$RhCl_2$]$_2$ (1 mol %), $AgSbF_6$ (10 mol %), NCTS (2.0 equiv), PhMe, 36 h, 120 °C	[37]
26.	*Phosphonate derivative*: [Cp*$RhCl_2$]$_2$ (5 mol%), $AgSbF_6$ (15 mol%), NCTS (2.0 equiv), DCE, 24h, 110°C	[38]

27.	*Diazo derivative*: [Cp*$RhCl_2$]$_2$ (5 mol %), $AgNTf_2$ (50 mol %),NaOAc (1.0 equiv), NCTS (1.5 equiv), DCE, 24h 130°C	[39]
28.	*N-Acylindolines*: [Cp*$RhCl_2$]$_2$ (5 mol %), $AgSbF_6$ (20 mol %), NCTS (2.0 equiv), NaOAc (30 mol %), DCE, 40 h, 130 °C<br>*N-Pyrimidyl Indoles*: [Cp*$RhCl_2$]$_2$ (5 mol %), $AgSbF_6$ (20 mol %), NCTS (2.0 equiv), NaOAc (30 mol %), DCE, 20 h, 110 °C	[40]
29.	*N-2-Pyridyl 3-carboxylic acid Indole derivative*: [Cp*$RhCl_2$]$_2$ (2.5 mol %), AgOAc (15 mol %), NCTS (2.0 equiv), MeOH, 24 h, 60 °C.	[41]
30.	*N-2-Pyridyl indole derivative*: [Cp*$RhCl_2$]$_2$ (1 mol %), $AgSbF_6$ (10 mol %), NCTS (1.0 equiv),^{t}AmOH, 18 h, 120 °C<br>*N-2-Pyridyl pyrrole derivative*: [Cp*$RhCl_2$]$_2$ (3 mol %), $AgSbF_6$ (10 mol %), NCTS (1.0 equiv), ^{t}AmOH, 18 h, 120 °C	[42]
31.	*Alkene derivative:* [Cp*$RhCl_2$]$_2$ (3 mol %), $Cu(OAc)_2$. H_2O (10 mol %), NCTS (2.0 equiv), ^{t}AmOH, 12 h, 120 °C	[43]
32.	*Acrylamide derivative*: RhCp*$(CH_3CN)_3(SbF_6)_2$ (10 mol %), Ag_2CO_3 (20 mol %), NaOAc (20 mol %), NCTS (2.0 equiv), DCE, 24 h, 120 °C	[44]
33.	*N-Methoxyamide derivative*: [Cp*$RhCl_2$]$_2$ (2.5 mol %), Ag_2CO_3 (100 mol %), NCTS (1.0 equiv), 1,4-dioxane, 8-16 h, 80 °C	[45]
34.	*N-Nitrosoamine derivative*: [Cp*$RhCl_2$]$_2$ (2.5 mol %), $AgSbF_6$ (15 mol %), H_2O (2.0 equiv), NCTS (1.5 equiv), Acetone; 24h 120°C	[46]
	3.2.3.1.2 Cyanation under Cobalt Catalysis	
35.	*2-Phenylpyridine derivative:* [Cp*CoI_2(CO)](2.5 mol %), $AgSbF_6$ (5 mol %), NCTS (1.5 equiv), KOAc (5 mol %), DCE, 16 h, 120 °C	[47]
36.	*2-Isopropenyl pyridine derivative:* [Cp*CoI_2(CO)](10 mol %), $AgSbF_6$ (20 mol %), NCTS (1.5 equiv), NaOAc (5 mol %), DCE, 24 h, 110 °C	[48]
37.	*Organozinc derivative*: NCTS (1.0 equiv), Zn dust, CH_3CN, 2-6 h, 0-50 °C	[49]
38.	*2-Arylpyridine derivative*: [Cp*CoI_2(CO)](10 mol %), $AgSbF_6$ (20 mol %), NCTS (1.0 equiv), DCE, 16 h, 120 °C	[50]
	**3.2.3.1.3 Cyanation under ruthenium catalysis**	
39.	*N,N-Dialkylamide derivative*: [$RuCl_2$ (*p-cymene*)]$_2$ (5 mol %), $AgSbF_6$ (20 mol %), NCTS (2.0 equiv), NaOAc (20 mol %), DCE, 24 h, 120 °C	[51]
40.	*Azaindole derivatives*: [$RuCl_2$ (*p-cymene*)]$_2$ (5 mol %), AgOTf (30 mol %), NCTS (2.0 equiv), NaOAc (50 mol %), DCE, 30 h, 110 °C	[52]
	**3.2.3.1.4 Cyanation under copper catalysis**	

41.	*2-Vinylnapthalene derivative*: [CuCl(Ligand 3)](20 mol %), $(BPin)_2$ (1.1-1.2 equiv), NCTS (1.2 equiv), LiO^tBu (1.5 equiv), 1,4-dioxane, 12 h, 80 °C	[53]
42.	*Styrene derivative*: IMesCuCl (10 mol %), $(BPin)_2$ (1.5 equiv) NCTS (2.0 equiv), LiO^tBu (2.0 equiv), THF, 4Å mol. sieve; 12 h, RT	[54]
43.	*Terminal allene*: ICyCuCl (10 mol %), $(BPin)_2$ (2.2 equiv), NCTS (1.5 equiv), NaO^tBu (2.2 equiv), THF, 40 h, RT	[55]
44.	*Dialkylnapthylene derivative:* [CuCl(Ligand 3)] (20 mol %),$(BPin)_2$ (1.1 equiv), NCTS (1.2 equiv), LiO^tBu (1.2 equiv), 1,4-dioxane, 12 h, 120 °C	[56]
	**3.2.3.1.5 Cyanation under palladium catalysis**	
45.	*Arenediazonium tetrafluoroborate or aryl halde:* $Pd(OAc)_2$ (15 mol %), Ag_2CO_3 (5 mol %), NCTS (2.0 equiv), EtOH, 15 h, 55-60 °C	[57]
	**3.2.3.2 Metal free electrophillic cyanation**	
46.	*With ketone:* i) *B*-Iodo-9-BBN *(1.0 equiv), i*Pr_2Net (1.0 equiv), Et_2O, 1 h, 0 °C-RT ii) NCTS (1.0 equiv), THF, 12 h, RT<br><br>*With α,β-unsaturated ketone:* i) 9-BBN (1.0 equiv), THF, 3 h, RT; ii) NCTS (1.0 equiv), THF, 12 h, RT	[59]
	**3.3. Cyanamides in radical reactions**	
47.	*With phenylselenide derivitised cyanamide*: Bu_3SnH/AIBN (2/1.5 equiv, 0.06 mmol/h), C_6H_6; reflux	[60]
48.	*With N-Acyl cyanamide alkene derivative*: Ph_2HPO (1.0 equiv), $AgNO_3$ (1.0 equiv), CH_3CN; Argon, 10h, 80°C	[63]
49.	*With boronic acid derivative*: PIFA (2.0 equiv), NBS (2.0 equiv), NH_2CN (1.1 equiv), CH_3CN, NaOH (aq), 1 h, RT	[64]
	**3.4. Co-ordination chemistry of cyanamides**	
	**3.4.1. Dialkylcyanamide complexes**	
50.	*Iron co-ordination complex*:<br><br>*With $FeCl_2$*: i) $P(OEt)_3$(2.0 equiv), EtOH; 1.5h reflux; ii) Et_2NCN (2.0 equiv), EtOH, 3 h, RT; iii) $NaBPh_4$ (excess), EtOH<br><br>Osmium/Ruthenium Hydride:<br><br>*With $[OsCl_6][NH_4]_2$ or $RuCl_3.3H_2O$*: i) $P(OEt)_3$ (10 equiv.), EtOH, RT-(50-60) °C, N_2; ii) $NaBH_4$ (13 equiv.), EtOH, 50-60 °C, N_2<br><br>Osmium cyanamide complex:<br><br>*With $[OsH_2(P(OEt)_3)_4]$* i) $CH_3OSO_2CF_3$ (1.0 equiv), toluene, 1h (-196 °C)–RT, N_2; ii) Et_2NCN (3.0 equiv,) in 2 mL EtOH, toluene, 2 h, RT, N_2; iii) $NaBPh_4$ (3.0 equiv), EtOH, RT-(-25 °C)	[69a]

	Ruthenium cyanamide complex:<br><br>*With [$RuH_2(P(OEt)_3)_4$]* i) (a) $HOSO_2CF_3$ (1.0 equiv.) Toluene; 1h (-196°C)–RT, N_2; (b) $HOSO_2CF_3$ (1.0 equiv.) toluene, 1 h (-196 °C)–RT, N_2; ii) Et_2NCN (3.0 equiv) in 2mL EtOH, toluene, 2 h, RT, N_2; iii) $NaBPh_4$ (3.0 equiv), EtOH, RT-(-25 °C)	
51.	*With $CoX_2.6H_2O$ (X=Cl, Br)*: Me_2NCN (2.0 equiv.), MeOH, 2 h, 50 °C	[70]
	**3.4.2. Aryl cyanamide complexes**	
52.	*With $Ni(OAc)_2.4H_2O$*: 4-nitrophenyl cyanamide (2.0 equiv), 1,10-phenanthroline (2.0 equiv), MeOH, 5 h, RT	[73]
53.	*With $Cd(OAc)_2$*: 4-nitrophenyl cyanamide (2.0 equiv), DMF, 3 h, RT	[74]
54.	*With 4-nitro/bromo/chlorophenyl cyanamide*:<br><br>i) NaOH (2.0 equiv), acetone, 12 h, reflux; ii) $Hg(NO_3)_2.H_2O$ (1.0 equiv), acetone, 4 h, reflux.	[75]
55.	Tin 4,4'-dicyanamide bipyridine complex:<br><br>*With $SnMe_3Cl$*: 4,4'-dicyanamidobiphenyl (0.5 equiv), NaOH (1.0 equiv), EtOH, 1 h, then 24 h sonication, RT<br><br>Tin 4-nitrophenyl cyanamide complex:<br><br>4-nitrophenyl cyanamide (0.5 equiv), NaOH (1.0 equiv), EtOH, 1 h, then 24 h sonication, RT	[76]
56.	Nickel Imidazole complex:<br><br>*With $Ni(OAc)_2.4H_2O$*: i) imidazole (4.0 equiv), MeOH, 3 h, RT; ii) 4-nitrophenyl cyanamide (2.0 equiv), MeOH, 4 h, RT<br><br>Nickel bipyridine complex:<br><br>*With $Ni(OAc)_2.4H_2O$*: i) 2,2'bipyridine (2.0 equiv), MeOH, 3 h, RT; ii) 4-nitrophenyl cyanamide (2.0 equiv), MeOH, 4 h, RT<br><br>Nickel Phenanthroline Complex:<br><br>*With $Ni(OAc)_2.4H_2O$*: i) 1,10-phenanthroline (2.0 equiv), MeOH, 3 h, RT; ii) 4-nitrophenyl cyanamide (2.0 equiv), MeOH, 4 h, RT	[77]
